# Soil and foliar Si fertilization alters elemental stoichiometry and increases yield of sugarcane cultivars

**DOI:** 10.1038/s41598-023-43351-z

**Published:** 2023-09-25

**Authors:** Alexson Filgueiras Dutra, Marcos Renan Lima Leite, Cíntia Carmen de Faria Melo, Danilo Silva Amaral, José Lucas Farias da Silva, Renato de Mello Prado, Marisa de Cássia Piccolo, Rafael de Souza Miranda, Gabriel Barbosa da Silva Júnior, Thâmara Kelly dos Santos Apollo Sousa, Lucas William Mendes, Ademir Sergio Ferreira Araújo, Alan Mario Zuffo, Francisco de Alcântara Neto

**Affiliations:** 1 Plant Science Department, Federal University of Piauí, Teresina, 64049-550 Brazil; 2 Postgraduate Program in Agronomy, São Paulo State University, Jaboticabal, 14884-900 Brazil; 3 Laboratory of Plant Nutrition, São Paulo State University, Jaboticabal, 14884-900 Brazil; 4 Center for Nuclear Energy in Agriculture, University of São Paulo, Piracicaba, 13416-000 Brazil; 5 Postgraduate Program in Agricultural Sciences, Federal University of Piauí, Bom Jesus, 64900-000 Brazil; 6 Department of Agricultural and Soil Engineering, Federal University of Piauí, Teresina, 64049-550 Brazil; 7 Department of Agronomy, State University of Maranhão, Balsas, MA 65800-000 Brazil

**Keywords:** Plant sciences, Element cycles

## Abstract

Silicon (Si) fertilization is widely recognized to improve the development of crops, especially in tropical soils and cultivation under dryland management. Herein, our working hypothesis was that Si stoichiometry favors the efficient use of carbon (C), nitrogen (N), and phosphorus (P) in sugarcane plants. Therefore, a field experiment was carried out using a 3 × 3 factorial scheme consisting of three cultivars (RB92579, RB021754 and RB036066) and three forms of Si application (control without Si; sodium silicate spray at 40 mmol L^−1^ in soil during planting; sodium silicate spray at 40 mmol L^−1^ on leaves at 75 days after emergence). All Si fertilizations altered the elemental C and P stoichiometry and sugarcane yield, but silicon-induced responses varied depending on sugarcane cultivar and application method. The most prominent impacts were found in the leaf Si-sprayed RB92579 cultivar, with a significant increase of 7.0% (11 Mg ha^−1^) in stalk yield, 9.0% (12 Mg ha^−1^) in total recoverable sugar, and 20% (4 Mg ha^−1^) in sugar yield compared to the Si-without control. In conclusion, our findings clearly show that silicon soil and foliar fertilization alter C:N:P stoichiometry by enhancing the efficiency of carbon and phosphorus utilization, leading to improved sugarcane production and industrial quality.

## Introduction

Silicon (Si) fertilization in sugarcane (*Saccharum officinarum* L.) has been cited to promote benefits in plant development, especially in tropical soils, which present low Si availability, and semiarid regions, which suffer drought episodes^1,2^. Silicon has also been cited to mitigate biotic and abiotic stresses that occur in crops, alleviating the damage to photosynthetic capacity and crop yield^3,4^.

The incorporation of Si into cell walls can occur as phytoliths^5^, a low-demand energy process compared to structural C compounds, suggesting a partial substitution of C for Si in some plant organic compounds, such as cellulose and hemicellulose^6^. In addition, absorbed Si can alter the elemental stoichiometric composition by increasing the metabolic efficiency of carbon (C) and the production of C skeletons, which favors the efficient utilization of nutrients, such as nitrogen (N) and phosphorus (P)^7,8^.

Silicon fertilization can also optimize the physiological aspects of sugarcane, an accumulator species, thus promoting plant growth and yield^9,10^. Within the plant, Si content alters the nutritional balance of several nutrients^11^, and internal or external factors that affect Si uptake can promote direct effects on stoichiometric modifications. Thus, factors such as Si application via soil or foliar spray, sugarcane cultivars and plant age can influence Si accumulation in plants^12^.

Previous studies showed disparity regarding the method of silicon application and absorption, particularly silicon uptake through roots compared to foliar absorption^13,14^. Thus, the researchers primarily concentrated on soil applications or nutrient solutions due to the argument that the role of silicon in roots would exert a more substantial physical impact on plants. In recent years, a prominent increase in studies focusing on foliar silicon application was noticed, highlighting that silicon not only has physical impacts but also promotes biochemical modulations in plants^15,16^.

A recent literature review^17^ evidenced that foliar silicon absorption holds significant relevance when supplementation is provided under optimal environmental conditions, such as moderate temperature and relative humidity. The authors emphasized that the amount of silicon absorbed by the leaves is lower than that absorbed by roots; nonetheless, foliar nutrition exhibits a more prominent “biochemical” signaling effect than the physical impact of root supply.

Recent investigations indicated that Si fertilization in sugarcane alters stoichiometric ratios (C:N, C:P, N:P and C:Si), with reduced C concentrations associated with increased N and P utilization and improved photosynthetic rates and biomass production^18,19^. Thus, studies of the stoichiometry of C, N, and P under Si application may reveal benefits to the management of Si fertilization in sugarcane crops to maximize the photosynthetic capacity of plants through increased homeostasis and efficiency of use of nutrients such as N and P, which are closely related to productivity^20^. However, evidence of how Si uptake in sugarcane is treated with different application methods (soil or foliar spray) and how different cultivars respond to Si fertilization under field conditions remain to be explored.

Our working hypotheses were that (i) Si fertilization and use vary according to the method of application in sugarcane cultivars; (ii) Si fertilization via soil, not foliar application, promotes modification of C:Si, C:N, C:P, and N:P stoichiometric ratios and improves biomass yield and technological quality of sugarcane. To test this hypothesis, field trials were carried out to examine whether Si supply via soil and foliar spray modifies C:Si:N:P stoichiometric ratios and improves the nutrient use efficiency and productivity of three sugarcane cultivars grown under dryland conditions.

## Materials and methods

### Location and characterization of the experimental area

**Figure 1:**
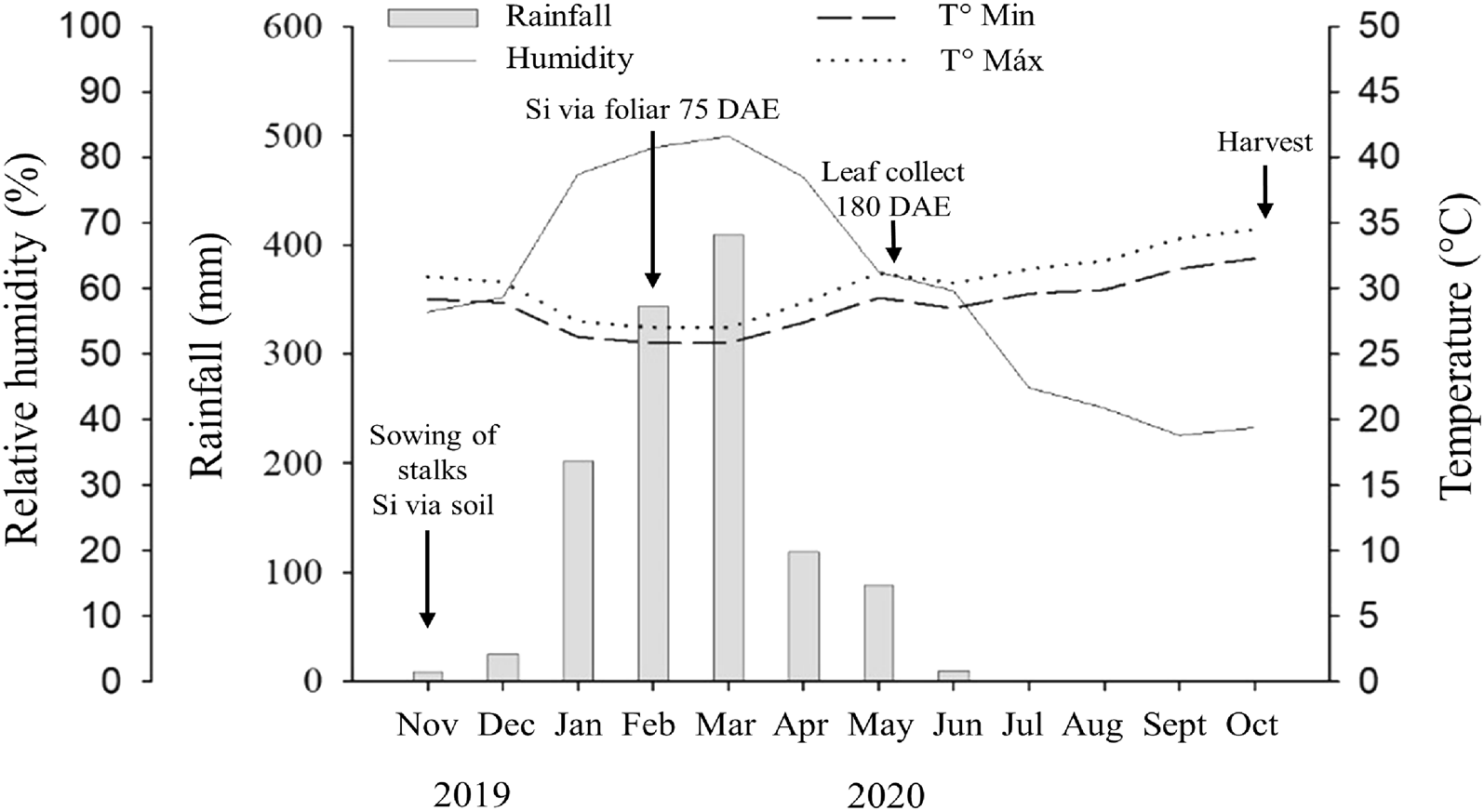
Rainfall, maximum and minimum air temperature, and humidity during the experimental period. *DAE* days after emergence.

The experiment was conducted in the 2019/20 harvest in a commercial sugarcane production area of Usina COMVAP—Açúcar e Álcool Ltda. (04° 52′ 56′′ S, 42° 52′ 58′′ W and altitude of 52 m), located in the municipality of União, Piauí, in the Mid-North region of Brazil. The regional climate is characterized as dry tropical, with an annual average temperature of 30 °C, featuring a dry winter and experiencing significant water scarcity from July to December^21^. The meteorological data during the trials were collected from a weather station installed near the experimental area (Fig. 1).

The soil of the experimental area is classified as an Oxisol^22^ or a dystrophic yellow Latosol of sandy loam texture. Soil samples were collected from 0 to 20 cm depth for chemical analysis of fertility^23^, and the following results were found: pH (H_2_O): 7.00; OM (organic matter): 11.00 g kg^−1^; P: 57.0 mg dm^−3^; K: 85.0 mg dm^−3^; Na: 10.0 mg dm^−3^; Ca: 2.72 cmol_c_ dm^−3^; Mg: 1.07 cmol_c_ dm^−3^; Al: 0.02 cmol_c_ dm^−3^; H + Al (potential acidity): 1.47 cmol_c_ dm^−3^; T (cation exchange capacity at pH 7.0): 5.52 cmol_c_ dm^−3^; and base saturation: 73%.

The available Si content in soil was determined according to Korndörfer^24^ and Crusciol et al.^25^. Briefly, 10 g of soil was homogenized in 100 mL of acetic acid extraction solution (0.5 mol L^−1^) and mixed at 240 rpm for 1.0 h. Subsequently, the material was kept in repose for 15 min and then filtered using quantitative filter paper. The Si content was measured in the supernatant via spectrophotometer readings at 660 nm, as described by Korndörfer^24^. Before planting, the Si concentration in the soil was found to be 6.0 mg dm^−3^.

### Treatments and experimental design

The treatments consisted of three sugarcane cultivars (RB92579, RB021754, RB036066 [all from the Interuniversity Network for Development of Sugar-Energy Sector]) and three forms of Si supply (no Si application [–Si]; soil-sprayed Si [SiS]; and foliar-sprayed Si [SiL]) in a factorial design (3 × 3) using a randomized block design with four repetitions. The experimental plots had an area of 84 m^2^ (six rows of 10 m), with 56 m^2^ of useful area (nett plot) to collect and evaluate the plants.

The area was prepared by plowing, harrowing, and opening furrows 40 cm deep and 1.4 m apart. The soil correction, planting and covering fertilizations were performed based on soil analysis and recommendations for the use of correctives and fertilizers^23^. Planting was performed manually considering an average of 18 buds per meter.

Sodium silicate (Na_2_SiO_3_) was used as the Si source at 40 mmol L^−1^, a chemical compound characterized by high solubility and containing 4.6% total Si and 3.7% highly available soluble Si. Silicon was sprayed once in the furrow bottom at planting time (SiS), while foliar application (SiL) was done at 75 days after emergence (DAE) with favorable weather conditions (temperature < 26 °C and relative humidity > 60%) at the time of application. A solution volume of 200 L ha^−1^ was used, and the pH was adjusted to 12.7 by adding NaOH to increase the availability of monomeric Si chemical species (H_4_SiO_4_). In the control treatments (–Si), water was applied to the plant. A knapsack sprayer with a pressure regulator was used, ensuring the homogeneity of ground distribution in all plots.

In all cases, the solution drip from foliar Si spray did not contribute to Si uptake by roots, as the applied volume was only enough to wet the leaves. Furthermore, no Si residues from foliar treatments contaminated the leaf Si quantification, as the treatments were applied at 75 DAE, and the assays were conducted at 180 and 340 DAE. This argument is supported by numerous rainfalls occurring between 75 and 180 DAE, which would have washed off the potential Si residues remaining on the leaf surface (Fig. 1).

### Leaf sampling

Leaf samples + 1 were collected at 180, and tissue from the central middle third of the leaf without the midrib was used for the analyses^24^. At 340 DAE, plants were harvested, and leaves were separated from the stalk. The samples were washed successively in tap water, neutral detergent solution (0.1% v:v), hydrochloric acid solution (0.1% v:v), and deionized water. The material was dried in an oven with forced air circulation at 65 ± 5 °C until reaching a constant mass to determine the dry mass. Subsequently, the material was ground in a Willey-type knife mill (Model TE-650).

### Variables analysed

#### Si, C, N and P contents

Silicon extraction from plant material was performed at 180 and 340 days after emergence (DAE) through alkaline digestion using H_2_O_2_ and NaOH solutions in an oven at 90 °C for 4.0 h. Subsequently, silicon was quantified through a colorimetric reaction with ammonium molybdate in an acidic environment (comprising oxalic acid and hydrochloric acid) by spectrophotometric readings at 410 nm^24^. Additionally, the carbon (C), nitrogen (N) and phosphorus (P) contents were also determined in leaves harvested at 180 and 340 DAE. The determination of C and N was performed by dry combustion (1000 °C) using an elemental analyzer (LECO truspec CHNS) calibrated with LECO standard 502–278 (C: 45.00% and N: 2.68%). Phosphorus was determined by the molybdenum antimony colorimetric method in a spectrophotometer^25^.

#### Stoichiometric ratios, use efficiency and nutrient accumulation

After measuring the Si, C, N, and P contents, the stoichiometric ratios C:Si, C:N, C:P, and N:P in sugarcane were calculated. For all cases, the data of leaf contents, dry mass at 340 DAE, and Si, C, N, and P accumulation were used to measure the use efficiencies of C, N, and P (CUE, NUE, and PUE, respectively), which is the quotient between leaf dry mass squared and nutrient accumulation^26^.

#### Biometric, technological and productivity parameters

The height of six plants in the nett plot (cm) was determined at harvest, at 340 DAE, by measuring with a tape from base to plant apex of the last fully expanded leaf. The sugarcane was manually harvested from the production area of each plot, and the cane yield per hectare (tons of cane/ha) was determined, with total fresh weight per plot being quantified with digital scales and subsequently estimated per hectare. Total recoverable sugar (Mg ha^−1^) and sugar yield (Mg ha^−1^) were determined according to the methodology proposed by Ref.^27^.

### Statistical analysis

The obtained data were submitted to analysis of variance by F test (α ≤ 5%), and the means were compared by Tukey’s test (p ≤ 5%). The analyses were performed using the statistical software SPEED Stat^28^.

### Ethics statement

The authors declare that this research was conducted ethically and responsibly, and all procedures were carried out according to relevant guidelines and regulations.

## Results

### Si, C, N and P contents of cultivars at 180 DAE

**Figure 2:**
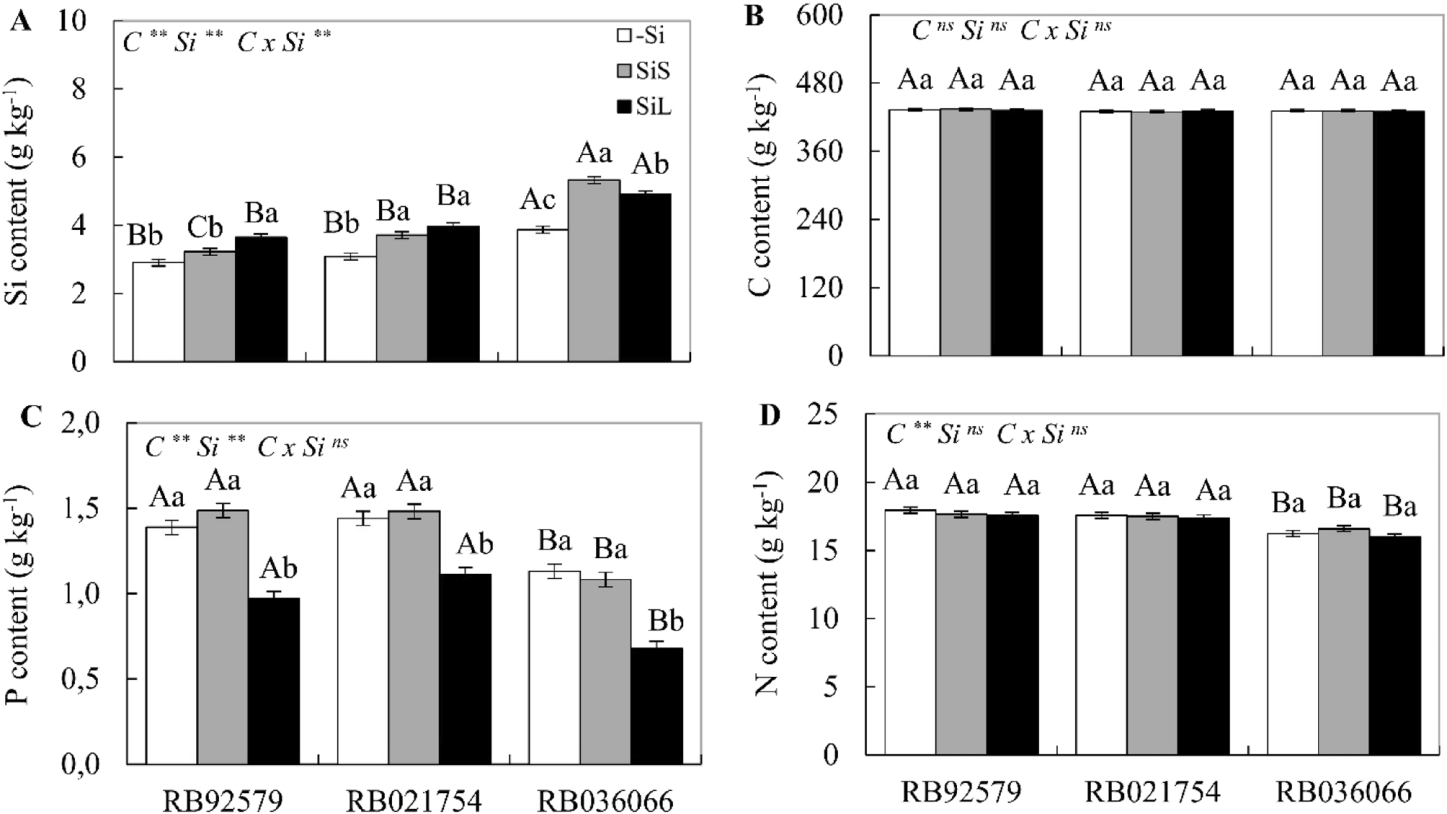
Silicon (**A**), carbon (**B**), nitrogen (**C**) and phosphorus (**D**) content in the leaves at 180 DAE in three sugarcane cultivars (RB92579, RB021754 and RB036066) under three forms of Si supply (without application of Si [–Si], Si via soil spray [SiS], and Si via foliar spray [SiL]). ** and * significant at 1 and 5% probability, respectively; *ns* nonsignificant by the F test. Lowercase letters show differences in Si forms, and uppercase letters show differences between cultivars by Tukey’s test. Bars represent the standard error of the mean, n = 4. Si × C: interaction.

Silicon application via soil or foliar spray resulted in a higher Si content in all cultivars, except for soil application to RB92579 (Fig. 2). In soil or foliar application, the Si content was higher in RB036066 than in the other sugarcane cultivars. Silicon application did not promote any alterations in the C and N contents of the sugarcane cultivars, but the P content decreased in all three cultivars under foliar application of Si (Fig. 2B–D).

The cultivars RB92579 and RB036066 showed the greatest increase in Si content when fertilized via foliar spray and soil, respectively, while there was no difference between the forms of Si supply for RB021754 (Fig. 2A). In the absence of Si, the cultivar RB036066 had the highest Si content and the lowest P and N contents, regardless of the Si supply (Fig. 2C,D), with no difference in C content between cultivars (Fig. 2B).

### Stoichiometric ratios C:N:P:Si of cultivars

**Figure 3:**
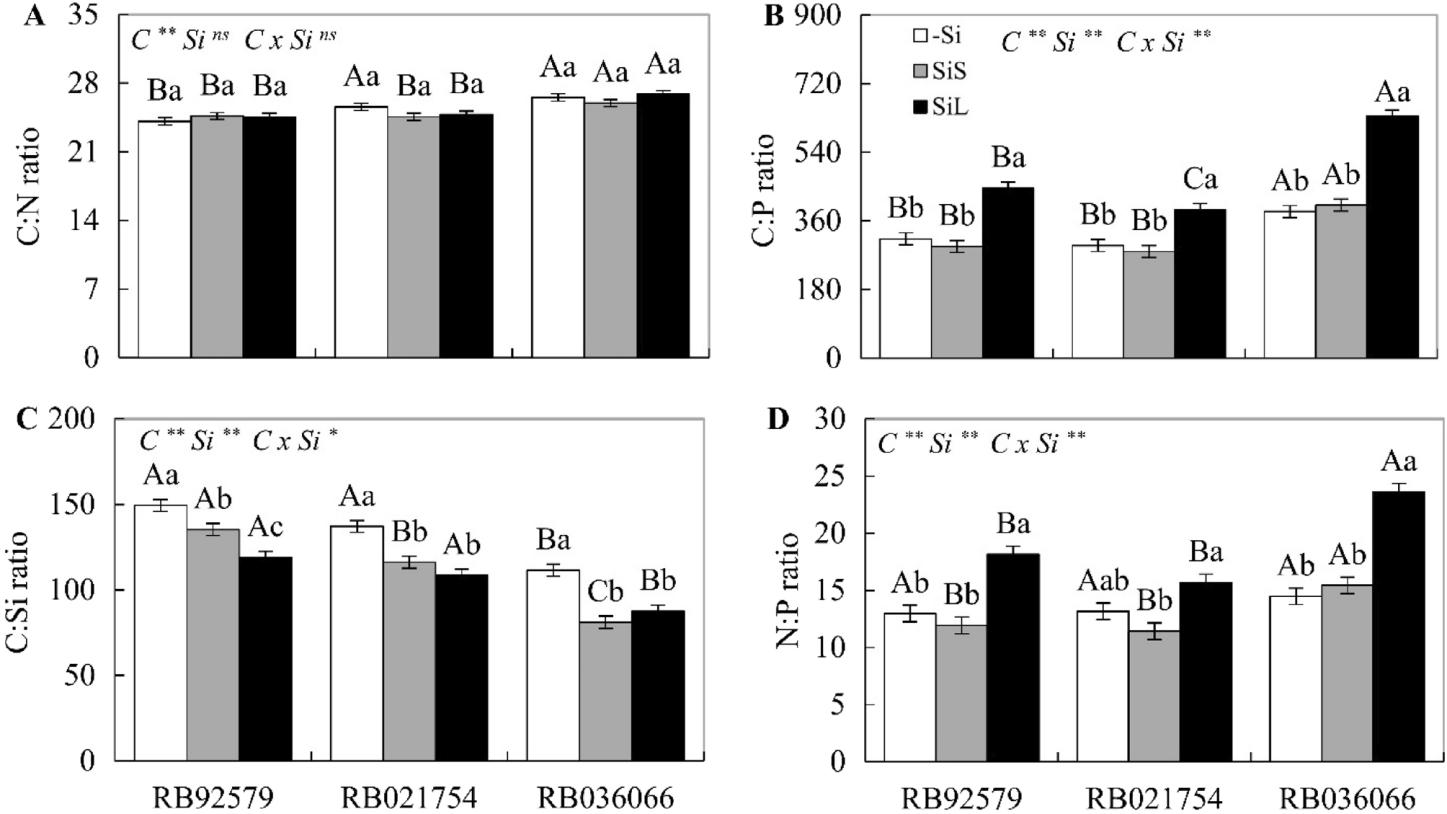
C:N ratio (**A**), N:P ratio (**B**), C:P ratio (**C**), and C:Si ratio (**D**) in the leaves at 180 days after emergence in three sugarcane cultivars (C) (RB92579, RB021754 and RB036066) under three forms of Si supply (without application of Si [–Si], Si via soil spray [SiS], and Si via foliar spray [SiL]). ** and * significant at 1 and 5% probability, respectively; *ns* nonsignificant by the F test. Lowercase letters show differences in Si forms, and uppercase letters show differences between cultivars by Tukey’s test. Bars represent the standard error of the mean, n = 4. Si × C: interaction.

In the absence of Si, the C:N, C:Si and C:P ratios were lower in cultivars RB92579, RB036066 and RB021754, respectively, without significant differences in the N:P ratio. In both soil and foliar Si applications, there was a reduction in the C:Si ratio of the three cultivars. Foliar fertilization with Si caused an increase in the C:P and N:P ratios of the three cultivars, with the highest values observed in RB036066, but the C:N ratio of the cultivars was not altered in the presence of Si (Fig. 3A–D).

In plants treated with Si via soil, the C:P ratio was lower in cultivars RB92579 and RB021754, and the C:N ratio was higher in cultivar RB036066 when applied via soil or foliar spray (Fig. 3A,B). In addition, there was a reduction in C:Si in all three cultivars, and the decrease in C:Si was more pronounced when Si was supplied via foliar application in RB92579 (Fig. 3C).

### C, N and P use efficiency

**Figure 4:**
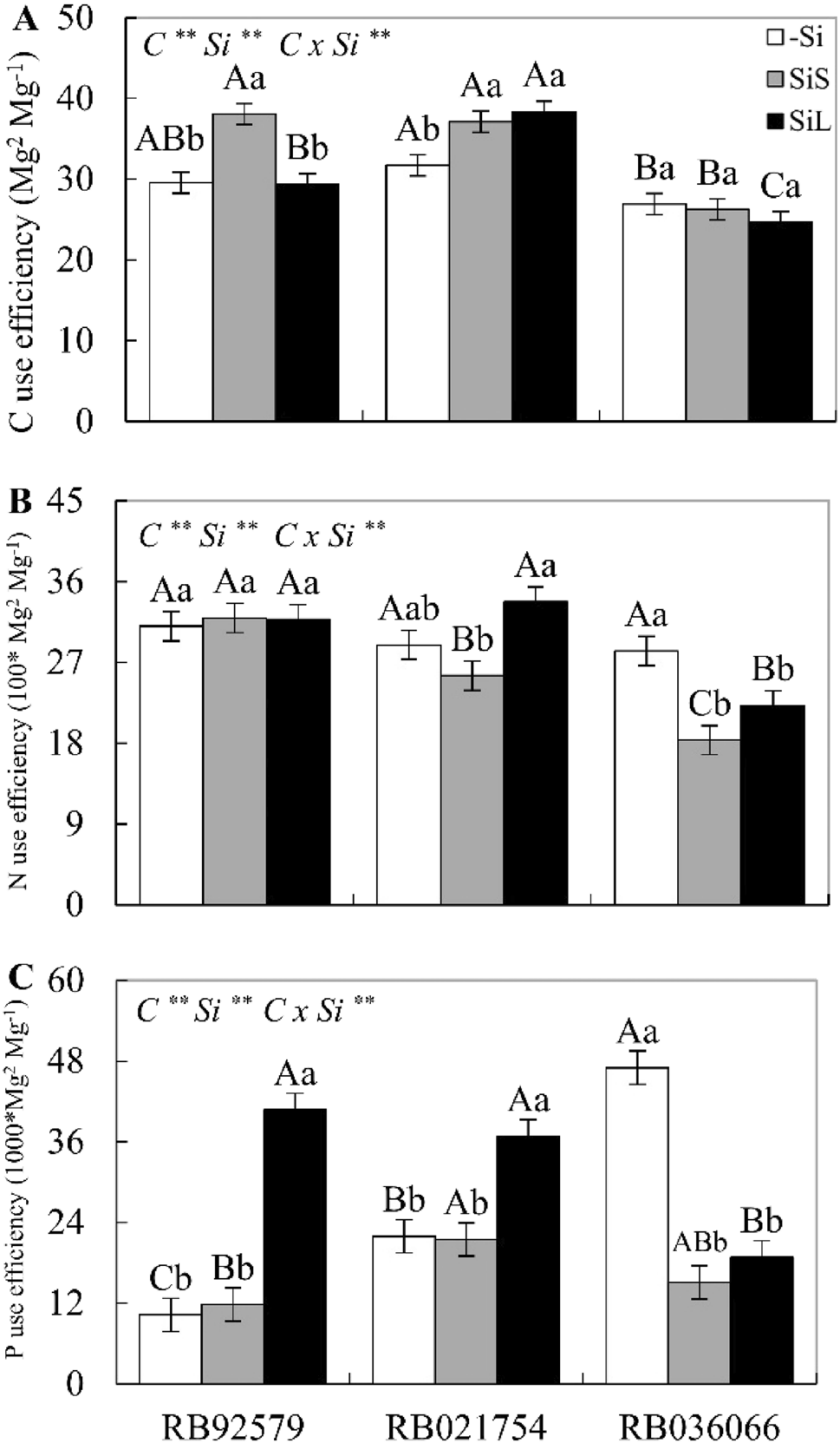
C use efficiency (**A**), N use efficiency (**B**), and P use efficiency (**C**) in the leaves at 340 DAE in three sugarcane cultivars (C) (RB92579, RB021754 and RB036066) under three forms of Si supply (without application of Si [–Si], Si via soil spray [SiS], and Si via foliar spray [SiL]). ** and * significant at 1 and 5% probability, respectively; *ns* nonsignificant by the F test. Lowercase letters show differences in Si forms, and uppercase letters show differences between cultivars by Tukey’s test. Bars represent the standard error of the mean, n = 4. Si × C: interaction.

In the absence of Si, cultivar RB036066 showed the highest P and lowest C use efficiency, while the highest C use efficiencies were recorded in cultivars RB92579 and RB021754. There were no differences among cultivars for N use efficiency (Fig. 4A–C). In contrast, in the presence of Si applied via soil, increased C use efficiency was observed in RB92579 and RB021754. Si-foliar-sprayed plants showed an increase in the use efficiency of C, N and P in the RB021754 cultivar and an increase in the use efficiency of P in RB92579 and RB021754 compared to those in the absence of Si. There was a decrease in N and P use efficiency in cultivar RB036066 under both forms of Si supply, with no change in C use efficiency (Fig. 4A–C).

### Accumulation of Si, C, N and P in sugarcane cultivars

**Figure 5:**
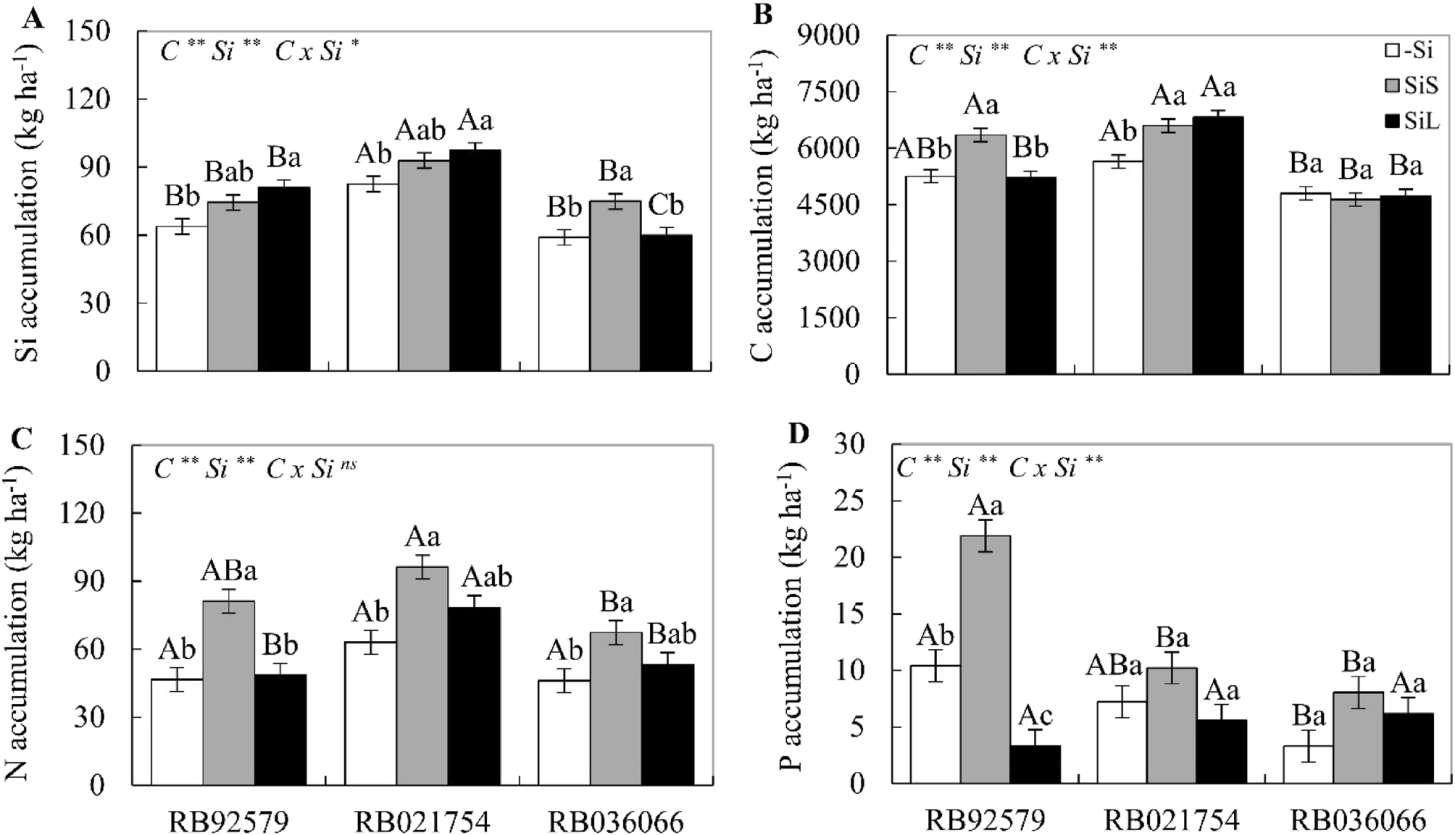
Silicon (**A**), carbon (**B**), nitrogen (**C**) and phosphorus (**D**) accumulation in the leaves at 340 DAE in three sugarcane cultivars (C) (RB92579, RB021754 and RB036066) under three forms of Si supply (without application of Si [–Si], Si via soil spray [SiS], and Si via foliar spray [SiL]). ** and * significant at 1 and 5% probability, respectively; *ns* nonsignificant by the F test. Lowercase letters show differences in Si forms, and uppercase letters show differences between cultivars by Tukey’s test. Bars represent the standard error of the mean. Si × C: interaction.

In the treatment without fertilization, Si accumulation was highest in RB021754, while RB92579 and RB021754 accumulated the most C and P, with no difference in N accumulation. In contrast, plants treated with Si in soil displayed an increase in the accumulation of Si and N in all three cultivars, as well as an accumulation of C in RB92579 and RB021754 and an accumulation of P in RB92579. Under foliar Si application, there was an increase in Si and C accumulation in RB021754 and an increase in Si accumulation and a decrease in P accumulation in RB92579 compared to untreated plants (Fig. 5A–D).

The cultivar RB021754 showed the highest accumulation of Si in all three forms of supply and the highest accumulation of N under soil and foliar Si fertilization. Additionally, under soil application, the cultivar RB92579 had an increase in N accumulation. The accumulation of C was higher in RB92579 and RB021754 and lower in RB036066, regardless of the presence or form of Si supply. Under foliar spray, RB021754 showed the highest accumulation of C. The accumulation of P was higher in RB92579 when Si was applied to the furrow but decreased when Si was applied to the leaves (Fig. 5A–D).

### Biometric, technological and productivity parameters

**Figure 6:**
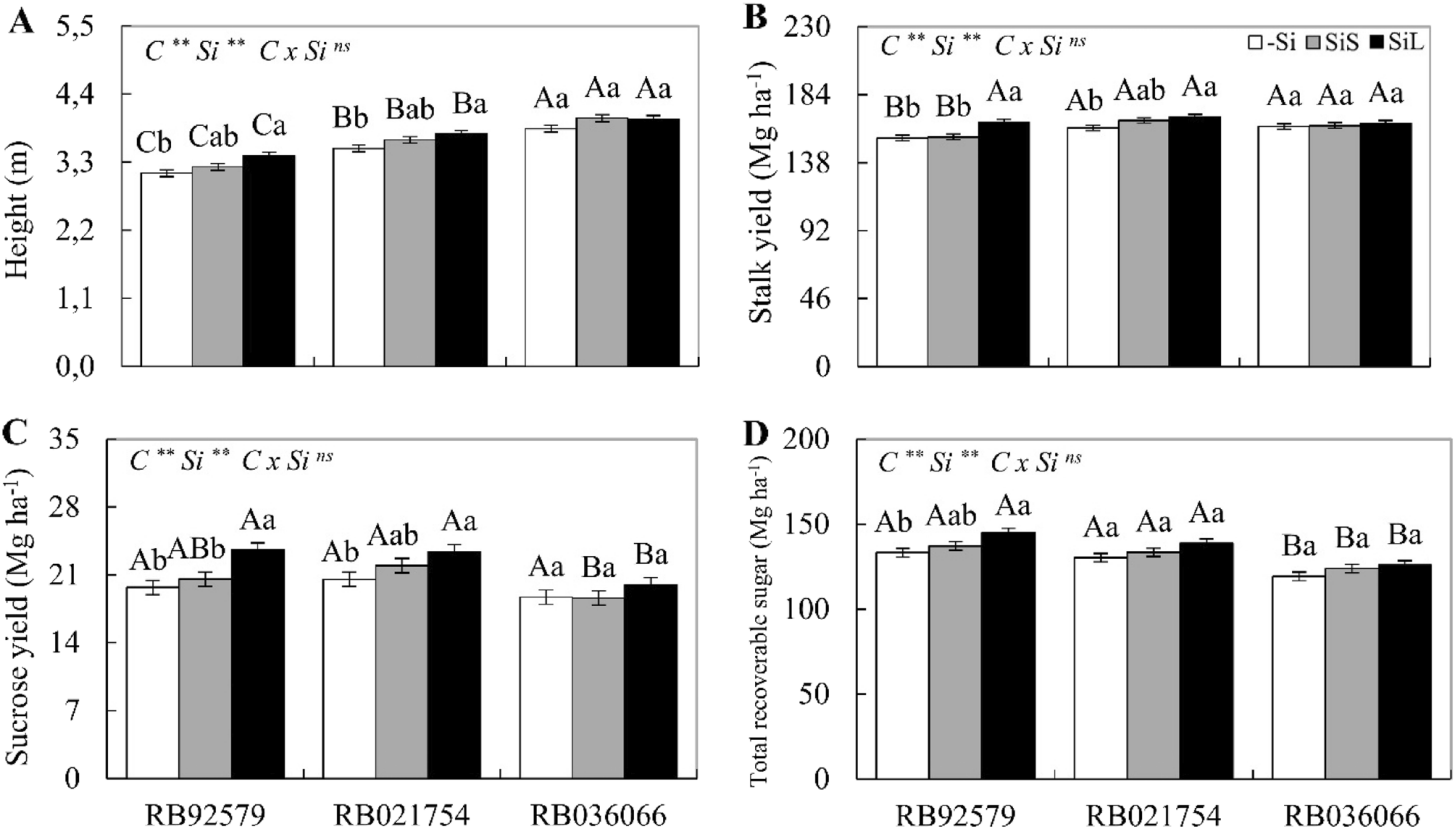
Plant height (**A**), stalk yield (**B**), sucrose yield (**C**), and total recoverable sugar (**D**) at 340 DAE in three sugarcane cultivars (C) (RB92579, RB021754 and RB036066) under three forms of Si supply (without application of Si [–Si], Si via soil spray [SiS], and Si via foliar spray [SiL]). ** and * significant at 1 and 5% probability, respectively; *ns* nonsignificant by the F test. Lowercase letters show differences in Si forms, and uppercase letters show differences between cultivars by Tukey’s test. Bars represent the standard error of the mean. Si × C: interaction.

In the absence of Si, the highest values of plant height were registered in cultivar RB036066, followed by RB021754 and RB92579, while the highest stalk yield occurred in cultivars RB021754 and RB036066 (Fig. 6A,B). There was no difference in sucrose yield between the studied cultivars, but cultivar RB03666 showed a lower total recoverable sugar value (Fig. 6C,D).

Silicon applied in soil increased the plant height of RB92579 and RB021754 cultivars, as well as the stalk and sucrose yield of RB021754 and total recoverable sugar in RB92579. Under foliar Si application, height, cane yield and sugar production increased in cultivars RB92579 and RB021754. In addition, an increase in total recoverable sugar was observed in cultivar RB92579. It is noteworthy that the cultivar RB036066 showed no significant effect of Si treatment on any of the yield parameters (Fig. 6A–D).

## Discussion

### Silicon modulates the nutritional status and accumulation of Si, C, N and P

The sugarcane crop is considered to accumulate Si that displays elevated rates of root Si uptake, a fact well documented in the literature^29^, but there are no convincing studies for foliar uptake. Our findings clearly indicated that foliar Si application is effective in increasing the Si content in the diagnostic leaf (leaf + 1), a response that depends on the form of application and sugarcane cultivar (Fig. 2A). These data validate the first hypothesis raised, highlighting that soil or foliar Si fertilization is effective in sugarcane grown in the field.

Silicon fertilization did not alter the N content, but when applied via foliar application, it significantly reduced the P content in plants. This argument was also previously described in maize and wheat plants fertigated with K_2_SiO_3_^30^ and confirmed in our field study, demonstrating that all three cultivars with or without foliar Si application showed P levels lower than the reference limit (1.5 to 3.0 g kg^−1^) adequate for sugarcane^31^. Phosphorus contents considered within the adequate range were observed only in cultivars RB92579 and RB021754 under soil Si fertilization (Fig. 2C).

The accumulation of Si and other nutrients also differed among cultivars. RB021754 exhibited a progressive increase in Si accumulation at levels similar to those of C accumulation. These findings suggest that Si supply increases the demand for carbon compounds for cell wall stabilization, mediating C incorporation into photosynthetically active tissues in association with stabilizing compounds^6^. Consequently, Si indirectly favored crop productivity, as sugarcane plants demand C for sucrose production and for respiration and cell wall synthesis^32^.

All three cultivars accumulated more N when treated with Si, and increased P accumulation was registered in the soil Si-fertilized RB92579 cultivar, indicating that sugarcane cultivars show different responses to Si fertilization^33^. Silicon can promote greater accumulation of C, N, and/or P in different cultivars, which depends on the combination of cultivar and form of silicon application.

### Silicon alters elemental stoichiometry, nutrient use efficiency and productivity in sugarcane

Elemental stoichiometry has been measured as a key mechanism of plant metabolism by considering nutrient conversion into plant biomass^19^. Previous reports demonstrated that Si might alter the C:N:P ratio and influence their accumulation and thus contribute to greater nutritional homeostasis^6,34^. In this study, sugarcane plants displayed different C:N, C:Si and C:P ratios as affected by Si fertilization. Both soil and foliar Si fertilization promoted a decreased C:Si ratio associated with higher Si content, while foliar Si fertilization induced increased C:P and N:P ratios in the three cultivars associated with lower P content.

Previous studies have demonstrated the beneficial role of foliar or soil Si application in stoichiometric modulations in sugarcane, especially at the seedling stage and/or in nutrient solution trials^35–38^. Our results now reinforce the stoichiometric modifications with Si via foliar or soil application in different sugarcane cultivars under field conditions^18,38^. A close relationship was observed between stoichiometric ratios and the C and P use efficiencies, with the highest and lowest C use efficiencies coinciding with the lowest (RB92579 and RB021754) and highest C:P ratios (RB036066), respectively (Figs. 3B, 4A,C). This regulation reveals that sugarcane cultivars use different mechanisms to establish nutritional homeostasis and thus convert nutrients into biomass.

Plants with high C:P ratios usually present limitations in carbon utilization since P is crucial for metabolic processes in plant cells^39–41^. In discordance, sugarcane plants showed an increase in the C:P ratio, especially under foliar Si spray, and no impairment was reported for C use efficiency. The data indicate that the cultivars RB92579 and RB021754 most likely increased their P use efficiency under soil and foliar Si application due to gains in C use efficiency.

The beneficial effects of Si supply during sugarcane cultivation in tropical environments are reflected in the productivity of cultivars^19,29^. Silicon application, via soil or foliar application, promoted better growth performance for RB92579 and RB021754 cultivars, as shown by increased plant height, as well as higher production of stalks and sugar. The gain in yield parameters was associated with greater use efficiency of C and P in the presence of Si, as reflected by the regulation of the C:Si and C:P ratios. However, although the RB92579 cultivar exhibited increases in all productive parameters in response to Si fertilization, cane production was found to increase only under foliar Si fertilization.

The cultivar RB021754 showed improvement in most of the technological parameters in the two forms of Si application, except for total recoverable sugars, while RB036066 plants did not show any productivity gains. Thus, the RB036066 cultivar most likely seemed to be insensitive to Si application because it had a lower N use efficiency and unaltered P use efficiency in response to an increased C:P ratio, in comparison to the others, which may compromise C assimilation^42,43^ and, consequently, the gain in sugarcane productivity^42^.

In general, silicon nutrition has been shown to modulate several physiological and biochemical pathways in various plant species, particularly by activating critical responses to mitigate damage caused by both abiotic and biotic stresses^43–45^. Increased silicon-mediated cell wall deposition helps reduce water loss through transpiration and mediates osmoregulatory responses, along with proline modulation, to adjust root hydraulic conductivity^46^. Silicon also optimizes thylakoid membrane protein components, enhancing the photosynthetic efficiency of rice plants under drought conditions^47^. Furthermore, leaf silicon spray may activate enzymatic and nonenzymatic antioxidant defense systems to counteract oxidative damage in leaf cells, thus maintaining photosynthetic pigments and photosynthetic performance^45,48,49^. These findings provide clear evidence that silicon induces metabolic responses that enhance a plant's potential performance.

In the current study, Si fertilization was effective using a soluble source applied to the leaves that favored the production of stalks and sugar in the RB92579 cultivar. Concordantly, this cultivar was tested under fertilization with steel slag but displayed no increase in the industrial variables^50^, most likely due to the low availability of the employed Si source. Therefore, the soluble source (Na_2_SiO_3_) via the leaves can be a promising source to contribute to greater benefits in sugarcane crops. Taken together, our findings partially validate the tested hypotheses, highlighting that soil and foliar Si fertilization promotes stoichiometric modifications that increase C and P use efficiencies and yield parameters of two cultivars fertilized with Si but has no effects on the C:N ratio and N use efficiency.

## Conclusion

Our findings show that the application of silicon, whether through soil or foliar fertilization, alters the C:N:P stoichiometry and improves the utilization of carbon and phosphorus in field-grown sugarcane plants. Employing a soluble silicon source emerges as a pragmatic and environmentally friendly approach to enhance sugarcane production while also increasing total recoverable sugar and sugar yield. The success of silicon application strategies varies depending on the particular sugarcane variety and the chosen application method.

## Data Availability

The authors confirm that the data supporting the findings of this study are available within the article.

## References

[1] Camargo MS, Bezerra BKL, Holanda LA, Oliveira AL, Vitti AC, Silva MA (2019). Silicon fertilization improves physiological responses in sugarcane cultivars grown under water deficit. J. Soil Sci. Plant Nutr..

[2] Teixeira GCM, Prado RM, Rocha AMS, Piccolo MC (2022). Silicon as a sustainable option to increase biomass with less water by inducing carbon: nitrogen: phosphorus stoichiometric homeostasis in sugarcane and energy cane. Front. Plant Sci..

[3] Ali N, Réthoré E, Yvin JC, Hosseini SA (2020). The regulatory role of silicon in mitigating plant nutritional stresses. Plants.

[4] Majumdar S, Prakash NB (2020). An Overview on the potential of silicon in promoting defense against biotic and abiotic stresses in sugarcane. J. Soil Sci. Plant Nutr..

[5] Singh P, Kumar V, Sharma A (2023). Interaction of silicon with cell wall components in plants: A review. J. Appl. Nat. Sci..

[6] Xia S, Song Z, Van Zwieten L, Guo L, Yu C, Hartley IP, Wang H (2020). Silicon accumulation controls carbon cycle in wetlands through modifying nutrients stoichiometry and lignin synthesis of *Phragmites australis*. Environ. Exp. Bot..

[7] Krouk G, Kiba T (2020). Nitrogen and phosphorus interactions in plants: From agronomic to physiological and molecular insights. Curr. Opin. Plant Biol..

[8] Wang J, Wen X, Zhang X, Li S, Zhang DY (2018). Coregulation of photosynthetic capacity by nitrogen, phosphorus and magnesium in a subtropical Karst forest in China. Sci. Rep..

[9] Hurtado AC (2020). Silicon application induces changes C:N:P stoichiometry and enhances stoichiometric homeostasis of sorghum and sunflower plants under salt stress. Saudi. J. Biol. Sci..

[10] Lata-Tenesaca LF, Prado RM, Piccolo MC, Silva DL, Silva JLF (2021). Silicon modifies C:N:P stoichiometry, and increases nutrient use efficiency and productivity of quinoa. Sci. Rep..

[11] Rocha JR, Prado RM, Piccolo MC (2022). New outcomes on how silicon enables the cultivation of Panicum maximum in soil with water restriction. Sci. Rep..

[12] Camargo MS, Keeping MG (2021). Silicon in sugarcane: Availability in soil, fertilization, and uptake. Silicon.

[13] Tubana BS, Babu T, Datnoff LE (2016). A review of silicon in soils and plants and its role in US agriculture: History and future perspectives. Soil Sci..

[14] Tripathi P, Subedi S, Khan AL, Chung YS, Kim Y (2021). Silicon effects on the root system of diverse crop species using root phenotyping technology. Plants.

[15] Shen Z, Pu X, Wang S, Dong X, Cheng X, Cheng M (2022). Silicon improves ion homeostasis and growth of liquorice under salt stress by reducing plant Na^+^ uptake. Sci. Rep..

[16] Teixeira GCM, Mello Prado R, Rocha AMS (2022). Low absorption of silicon via foliar in comparison to root application has an immediate antioxidant effect in mitigating water deficit damage in sugarcane. J. Agro Crop Sci..

[17] Flores RA, Xavier MFN, de Mello Prado R (2023). Innovative sources and ways of applying silicon to plants. Benefits of Silicon in the Nutrition of Plants.

[18] Frazão JJ, Prado RM, Souza Júnior JP, Rossato DR (2020). Silicon changes C:N:P stoichiometry of sugarcane and its consequences for photosynthesis, biomass partitioning and plant growth. Sci. Rep..

[19] Oliveira Filho ASB, Prado RM, Teixeira GCM, Piccolo MC, Rocha MAS (2021). Water deficit modifies C:N:P stoichiometry affecting sugarcane and energy cane yield and its relationships with silicon supply. Sci. Rep..

[20] Wang J, Liu X, Zhang X, Li L, Lam SK, Pan G (2019). Changes in plant C, N and P ratios under elevated [CO_2_] and canopy warming in a rice-winter wheat rotation system. Sci. Rep..

[21] Leite MRL, Alcântara Neto F, Dutra AF, Mendes LW, Antunes JEL, Melo VMM, Oliveira FAZ, Rocha SMB, Pereira APA, Prado RM, Araújo ASF (2023). Silicon application influences the prokaryotic communities in the rhizosphere of sugarcane genotypes. Appl. Soil Ecol..

[22] *USDA Keys to Soil Taxonomy: Soil Conservation Service, 13th edition*. https://www.nrcs.usda.gov/sites/default/files/2022-09/Keys-to-Soil-Taxonomy.pdf (Accessed 10 December 2022) (2022).

[23] Cantarella H, Quaggio JA, Mattos Junior D, Boaretto RM, Van Raij B (2022). Boletim 100: Recomendações de adubação e calagem para o estado de São Paulo.

[24] Korndörfer GH, Pereira HS, Nolla A (2004). Análise de silício no solo, planta e fertilizantes.

[25] Crusciol CAC, Arruda DP, Fernandes AM, Antonangelo JA, Alleoni LRF, Nascimento CACD, Rossato OB, McCray JM (2018). Methods and extractants to evaluate silicon availability for sugarcane. Sci. Rep..

[26] Mabagala FS, Geng YH, Cao GJ, Wang LC, Wang M, Zhang ML (2020). Effect of silicon on crop yield, and nitrogen use efficiency applied under straw return treatments. Appl. Ecol. Environ. Res..

[27] Liu J, Basnayake J, Jackson PA, Chen X, Zhao J, Zhao P, Yang L, Bai Y, Xia H, Zan F, Qin W, Yang K, Yao L, Zhao L, Zhu J, Lakshmanan P, Zhao X, Fan Y (2016). Growth and yield of sugarcane genotypes are strongly correlated across irrigated and rainfed environments. Field Crops Res..

[28] Carvalho AMX, Mendes FQ, Mendes FQ, Tavares LF (2020). SPEED Stat: A free, intuitive, and minimalist spreadsheet program for statistical analyses of experiments. Crop Breed Appl. Biotechnol..

[29] Camargo MS, Korndörfer GH, Wyler P (2014). Silicate fertilization of sugarcane cultivated in tropical soils. Field Crops Res..

[30] Greger M, Landberg T, Vaculík M (2018). Silicon influences soil availability and accumulation of mineral nutrients in various plant species. Plants.

[31] Quaggio JA, Cantarella H, Quaggio JA, Mattos D, Boaretto RM, Raij B (2022). Cana-de-açúcar. Boletim 100: Recomendações de adubação e calagem para o estado de São Paulo.

[32] Wang J, Nayak S, Koch K, Ming R (2013). Carbon partitioning in sugarcane (*Saccharum* species). Front. Plant Sci..

[33] Camargo MS, Bezerra BKL, Vitti AC, Silva MA, Oliveira AL (2017). Silicon fertilization reduces the deleterious effects of water deficit in sugarcane. J. Soil Sci. Plant Nutr..

[34] Neu S, Schaller J, Dudel EG (2017). Silicon availability modifies nutrient use efficiency and content, C:N:P stoichiometry, and productivity of winter wheat (*Triticum aestivum* L.). Sci. Rep..

[35] Acreche MM (2017). Nitrogen-, water- and radiation-use efficiencies affected by sugarcane breeding in Argentina. Plant Breed..

[36] Santos LCN, Teixeira GCM, Prado RM, Rocha MAS, Pinto RCS (2020). Response of presprouted sugarcane seedlings to foliar spraying of potassium silicate, sodium and potassium silicate, nanosilica and monosilicic acid. Sugar Tech.

[37] Souza Júnior JP, Oliveira TL, Prado RM, Oliveira KR, Soares MB (2022). Analyzing the role of silicon in leaf C:N:P stoichiometry and its effects on nutritional efficiency and dry weight production in two sugarcane cultivars. J. Soil Sci. Plant Nutr..

[38] Teixeira GCM, Prado RM, Rocha MAS, Piccolo MC (2020). Root-and foliar-applied silicon modifies C:N:P ratio and increases the nutritional efficiency of presprouted sugarcane seedlings under water deficit. PLoS ONE.

[39] Prado RM (2021). Mineral Nutrition of Tropical Plants.

[40] Carstensen A, Herdean A, Schmidt SB, Sharma A, Spetea C, Pribil M, Husted S (2018). The impacts of phosphorus deficiency on the photosynthetic electron transport chain. Plant Physiol..

[41] Silva JLF, Prado RM (2021). Elucidating the action mechanisms of silicon in the mitigation of phosphorus deficiency and enhancement of its response in sorghum plants. J. Plant Nutr..

[42] Caione G (2015). Phosphorus fractionation in soil cultivated with sugarcane fertilized by filter cake and phosphate sources. Commun. Soil Sci. Plant. Anal..

[43] Patel M, Fatnani D, Parida AK (2021). Silicon-induced mitigation of drought stress in peanut genotypes (*Arachis hypogaea* L.) through ion homeostasis, modulations of antioxidative defense system, and metabolic regulations. Plant Physiol. Biochem..

[44] Zuffo AM, Ratke RF, Steiner F, Oliveira AM, Aguilera JG, Lima RE (2022). Silicon mitigates the effects of moderate drought stress in cover crops. J. Agron. Crop Sci..

[45] Leite WS, Miranda RS, Rocha MM, Dutra AF, Santos AS, Silva AC, Brito FM, Sousa RS, Araújo ASF, Nascimento CWA, Zuffo AM, Alcântara Neto F (2023). Silicon alleviates drought damage by increasing antioxidant and photosynthetic performance in cowpea. J. Agron. Crop Sci..

[46] Avila RG, Magalhães PC, Silva EM, Souza KRD, Campos CN, Alvarenga AA, Souza TC (2021). Application of silicon to irrigated and water deficit sorghum plants increases yield via the regulation of primary, antioxidant, and osmoregulatory metabolism. Agric. Water Manag..

[47] Wang Y, Zhang B, Jiang D, Chen G (2019). Silicon improves photosynthetic performance by optimizing thylakoid membrane protein components in rice under drought stress. Environ. Exp. Bot..

[48] Teixeira GCM, Prado RM, Rocha AMS, Oliveira Filho ASB, Sousa Junior GS, Gratão PL (2022). Action of silicon on the activity of antioxidant enzymes and on physiological mechanisms mitigates water deficit in sugarcane and energy cane plants. Sci. Rep..

[49] Xu J, Guo L, Liu L (2022). Exogenous silicon alleviates drought stress in maize by improving growth, photosynthetic and antioxidant metabolism. Environ. Exp. Bot..

[50] Sobral MF, Nascimento CW, Cunha KP, Ferreira HA, Silva AJ, Silva FB (2011). Basic slag and its effects on the concentration of nutrients and heavy metals in sugarcane. Rev. Bras. Eng. Agric. Ambient..

